# Study on Properties of Bone Glue/Polyurethane Composite Modified Asphalt and Its Mixture

**DOI:** 10.3390/ma14143769

**Published:** 2021-07-06

**Authors:** Wei Yan, Yangjia Ou, Jing Xie, Tuo Huang, Xinghai Peng

**Affiliations:** 1 Hunan Zhongda Design Institute Co., Ltd., Central South University, Changsha 410075, China; hnzdsjy@yeah.net (W.Y.); xianlusuo@yeah.net (Y.O.); 2 Key Laboratory of Highway Engineering of Ministry of Education, School of Traffic and Transportation Engineering, Changsha University of Science & Technology, Changsha 410114, China; xiejing@csust.edu.cn (J.X.); pengxinghsi@stu.csust.edu.cn (X.P.)

**Keywords:** modified asphalt, bone glue, polyurethane, asphalt mixtures, performance

## Abstract

Composite modification technology is widely used in the materials field. To enhance the property of polyurethane modified asphalt and realize its application in road engineering, the bone glue/polyurethane composite modified asphalt (CMA) was prepared using bone glue, polyurethane, and neat asphalt in this research. The bone glue content ranges 5–10%, that of the polyurethane is 1–5%. The relationship between the modifier’s content and the conventional properties and rheological properties of CMA was revealed by response surface methodology (RSM). The CMA performance was further verified under the optimal content of the bone glue and polyurethane. The differences of properties of styrene–butadienestyrene (SBS) modified asphalt mixture, neat asphalt mixture, and bone glue/polyurethane CMA mixture were compared and analyzed by using the pavement performance test. The results showed that the CMA’s conventional properties and rheological properties are improved. The optimal bone glue content and polyurethane content determined by RSM are 6.848% and 2.759%, respectively. The low-temperature crack resistance and water stability of the CMA mixture are enhanced, better than neat asphalt mixture and SBS modified asphalt mixture. The CMA mixture’s dynamic stability is 85% of SBS modified asphalt mixture, but it is 2.4 times of neat asphalt mixture. The result indicated that the bone glue/polyurethane CMA mixture still has certain advantages of high-temperature stability. In this research, the composite modification of bone glue and polyurethane can significantly enhance the characteristic of asphalt and asphalt mixture and provide a new method for applying and promoting polyurethane modified asphalt in road engineering.

## 1. Introduction

Globally, the asphalt pavement is widely applied in road engineering since its smooth surface, good driving comfort, and short construction period [1]. With the development of the global economy and the improvement of people’s living standards, the traffic load is increasing. Under the combined influence of natural environment, traffic load, and other factors, the rutting, potholes, and cracks frequently appear, which causes the pavement’s actual service life to be far shorter than the design life [2,3,4]. In order to meet the function requirements of petroleum asphalt in road engineering, modified asphalt can be prepared by adding certain modified materials to the asphalt [5,6]. This method dramatically improve the pavement performance of asphalt. Among them, polymer modifiers can increase the service life and service level of pavements which are widely used in road engineering [7,8,9].

The polymers modifiers can be divided into three categories according to their chemical structure and properties: plasticizers, elastomers, and reactive polymers [10]. The most widely used are SBS, polyethylene, and polypropylene. However, polymer-modified asphalt has poor compatibility and poor thermal storage stability [11,12]. Since the late 1960s, polyurethane has been widely used worldwide in coatings, elastomers, sealants, and other fields all over the world [13]. Polyurethane is a new type of organic polymer material with a two-phase structure [14]. Compared with plasticizers and elastomer polymer modified asphalt, polyurethane can improve asphalt’s technical properties and form a chemically cross-linked network structure system with asphalt, which has good storage stability. Some scholars have conducted tentative research work on the application of polyurethane in road engineering. Li et al. determined that the optimum shear temperature and shear time in the polyurethane modified asphalt preparation process were 120 °C and 10 min, respectively [15]. The results showed that polyurethane modified asphalt had wonderful low-temperature performance, but the water stability needs to be improved. Bazmara et al. found that polyurethane’s addition to asphalt can improve its low-temperature deformation ability [16]. It was inferred that polyurethane, as a modifier, reacts with asphalt to form new chemical bonds through infrared spectroscopy experiments. Khairuddin et al. used the response surface method to determine the optimal polyurethane content at 3% through a central composite design [17]. From the current research, polyurethane-modified asphalt can improve the asphalt’s low-temperature properties. However, how to improve the polyurethane modified asphalt’s high-temperature rheological properties is a problem that needs to be overcome.

At present, studies have shown that composite modification technology can effectively improve the performance of asphalt and its mixture, such as cooking oil/crumb tire rubber, rock asphalt/SBS, and rock asphalt/crumb rubber composite modified asphalt [18,19,20]. Jin et al. used rock asphalt to modify polyurethane modified asphalt [21]. They found that polyurethane improved the asphalt’ low-temperature properties, and rock asphalt improved the high-temperature properties. Bu et al. used the polyurethane/epoxy resin to prepare modified asphalt [22]. The experiment results showed that the low-temperature performance and elongation at break of the modified asphalt and the mixture’s high-temperature rutting resistance could be improved. Yu et al. used polyurethane and nano-graphene oxide to prepare composite modified asphalt and concluded that the mixture’s pavement performance was enhanced [23]. The reason is that the synergistic effect of nano inorganic filler and polyurethane improves the elastic modulus of neat asphalt. The yield strength of the asphalt mixture can also be enhanced after adding polyurethane. This compounding scheme improves the modulus of modified asphalt and maintains the toughness of the mixture. The feasibility of compound modified asphalt technology can be found based on the existing research [24,25,26]. However, the practical application of modifiers such as epoxy resin and nano-graphene oxide will be limited by their high prices [27,28]. Suppose we want to promote and apply this technology in road engineering comprehensively. In that case, it is necessary to look for a cheap and easy-to-obtain material to compound the asphalt with polyurethane.

Bone glue is a protein-based colloid made from collagen extracted from animal bones, leather, and meat waste. As a biodegradable water-soluble natural adhesive, bone glue is widely used in the wood industry and packaging industry [29,30]. Bone glue is extracted from organic waste, which is harmful to the environment. The application of bone glue in road engineering can enhance the pavement property of asphalt and its mixture and realize the recycling of bone glue materials, which has excellent economic and environmental benefits. Ye et al. found that bone glue can increase the viscosity of neat asphalt and enhance its elastic recovery ability under load [31]. Besides, bone glue can also improve the high-temperature shear deformation resistance. Rizvi et al. found that bone glue modified asphalt has better resistance to fuel solubility and can be applied to airport roads [32]. However, the low-temperature rheological characteristic of the bone glue modified asphalt and the low-temperature cracking resistance performance of its mixture need to be further improved [33].

To sum up, the bone glue and polyurethane were mixed into neat asphalt to prepare bone glue/polyurethane composite modified asphalt (CMA), so as to give full play to their respective technical characteristics and advantages, so that the CMA has better high-temperature and low-temperature flow. The test scheme can be determined by the miscellaneous response surface design. Different bone glue and polyurethane content on the conventional performance and rheological properties were studied. Through the comprehensive evaluation of the CMA performance, the optimal content of bone glue and polyurethane was determined. Finally, the pavement performance of neat asphalt mixture, styrene–butadienestyrene (SBS) modified asphalt mixture, and bone glue/polyurethane CMA mixture was studied to verify the feasibility of the compound technology of the bone glue and polyurethane. In this research, the composite modification of bone glue and polyurethane can significantly enhance the characteristic of asphalt and asphalt mixture and provide a new method for applying and promoting polyurethane modified asphalt in road engineering. On the other hand, the development of this study has realized the comprehensive and efficient use of bone glue. It can reduce environmental pollution.

## 2. Materials and Test Methods

### 2.1. Materials

#### 2.1.1. Neat Asphalt

In this research, the Liaohe A-70 was used as the neat asphalt, and its primary performance was tested according to JTG E20-2011. It can be seen from Table 1 that the performance of neat asphalt meets the requirements of the specification.

#### 2.1.2. Bone Glue

The bone glue used in this study is an industrial grade material from the Hebei Dongsheng beeswax factory, Hebei, China. According to the literature research, bone glue was treated with aluminum sulfate, urea, phenol, and deionized water [34,35,36]. The test method is as follows. The bone glue is dissolved in water in a beaker according to the water–cement ratio of 1.2:1. The beaker is then put into a constant temperature water bath, and then 7% aluminum sulfate, 3% urea, and 1% phenol are added into the beaker successively [31]. After 30 min of stirring, the light-yellow viscous glue liquid is obtained, which is the new bone glue aqueous solution. For the convenience of expression, the treated bone glue is still called bone glue in this paper. 

#### 2.1.3. Polyurethane

The polyurethane was produced by Badische Anilin-und-Soda-Fabrik (BASF). The ratio of the soft segment to the hard segment is 6:4. The test results are shown in Table 2.

#### 2.1.4. Aggregates and Filler

Coarse aggregate should be choosing the gravel, which is hard, wear-resistant, no weathering, and no impurities, dry and clean surface because there is good adhesion between asphalt and these coarse aggregates. In this research, the coarse aggregate used limestone, and the fine aggregate was limestone parent rock. The technical properties and specifications of aggregate are shown in Table 3.

### 2.2. Test Design

In this paper, the Miscellaneous response surface method (RSM) was selected to design the experiment. The contents of bone glue and polyurethane were taken as independent variables, and the conventional properties and rheological properties of asphalt were used as evaluation indexes. According to the existing research and previous test, the content range of bone glue is chosen to be 5–10%, and that of polyurethane is chosen to be 1–5% [21,29,31]. Design expert 10.0 was used for design, and Table 4 is the contents of bone glue and polyurethane.

#### 2.2.1. Preparation of the CMA

According to the bone glue and polyurethane contents in Table 4, 13 groups of CMA were prepared. The specific preparation process is as follows. The neat asphalt was heated in an oven at 135 °C for four hours, and then the neat asphalt was quickly transferred to the electric furnace for continuous heating and heat preservation. Meanwhile, the temperature was stable at 135 °C. The prepared bone glue was slowly and batched, mixed into the neat asphalt, and was added several times and manually stirred for 5 min until no obvious bubble was produced. Then polyurethane was added and mixed in the asphalt. After stirring for 5 min, it was sheared by high-speed shear apparatus. The shearing temperature was 145 °C, the shearing time was 40 min, and the shear rate was 3000 r/min. The electric furnace should be turned off after 40 min. The remaining heat was used to mix the modified asphalt manually. After cooling, bone glue/polyurethane CMA can be obtained.

#### 2.2.2. Mix Design of the CMA Mixture

**Figure 1:**
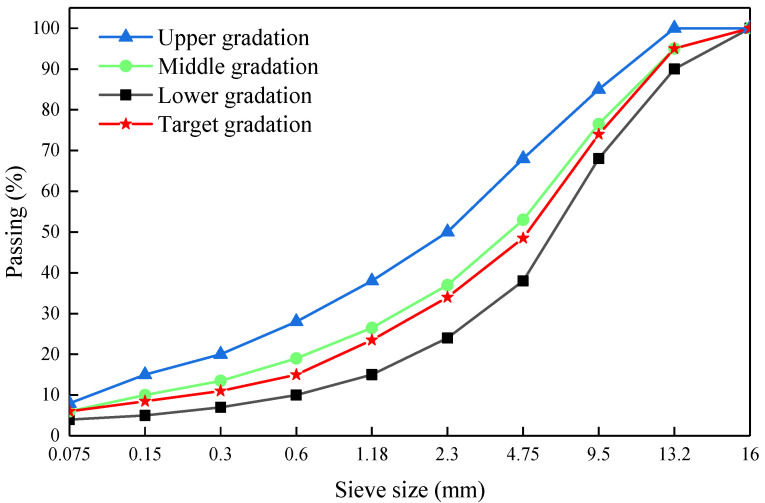
Gradation curve of mixture.

The gradation range of CMA mixture is determined according to the climate, traffic conditions, and highway grade, which conforms to the engineering design. In this research, according to the grading interval required by AC-13 asphalt mixture, which is widely applied in the practical application of freeway asphalt pavement, the gradation curve of asphalt mixture is obtained according to the design requirements, as shown in Figure 1.

### 2.3. Test Schemes

#### 2.3.1. Physical Properties Test of Asphalt

The penetration tests were carried out by CXS-2801 Penetration Tester produced by Shanghai Changji Geological Instrument Co., Ltd. Shanghai, China in this research, according to ASTM D5. Penetration test results only represent the soft and hard degree of asphalt, a basic performance test. The ductility test and softening point test at 5 °C were conducted to study CMA’s physical properties by SYD-2806F Softening Point Tester produced by Shanghai Changji Geological Instrument Co., Ltd. based on the ASTM D36 and ASTM D113, respectively.

#### 2.3.2. Multiple Stress Creep Recovery Test (MSCR)

Based on the AASHTO T350 and ASTM D 7405 test standards, the MSCR test of CMA was carried out by the MCR302 dynamic shear rheometer produced by Anton Paar, Hobart, Austria. The test conditions are as follows. In the stress-recovery mode of DSR, the CMA was loaded for 1 s and then unloaded for 9 s. At this time, the whole test temperature was controlled at 60 °C. In the test process, the stress of 1.0 kPa was applied to the specimen, and the cycle was repeated 10 times. The stress of 3.2 kPa was then applied to the specimen and repeat the cycle 10 times. Finally, the non-recoverable creep compliance was measured.

#### 2.3.3. Bending Beam Rheometer Test (BBR)

**Figure 2:**
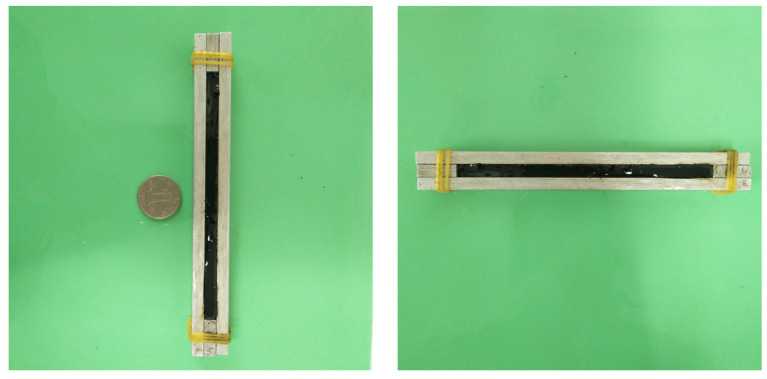
Specimen of the bending beam rheometer test.

In this research, the BBR test was conducted curved beam rheometer produced by Canon Instruments, Shanghai, China, and the test temperature was −18 °C. The rolling thin film oven test and pressure aging vessel test were carried out before conducting the BBR test. BBR test can be used to measure the stiffness of asphalt beam under creep load. When the temperature of pavement decreases, the gradually accumulated stress can be simulated by creep load. The parameters of load, deformation, creep rate, and creep stiffness at the 60 s can be automatically collected and calculated by the computer data acquisition system. Asphalt’s capacity to resist load is indicated by creep stiffness and the change rate of asphalt stiffness under load, expressed by *S*, and *m*, respectively. The Figure 2 is the specimen of the bending beam rheometer test.

#### 2.3.4. High-Temperature Stability

**Figure 3:**
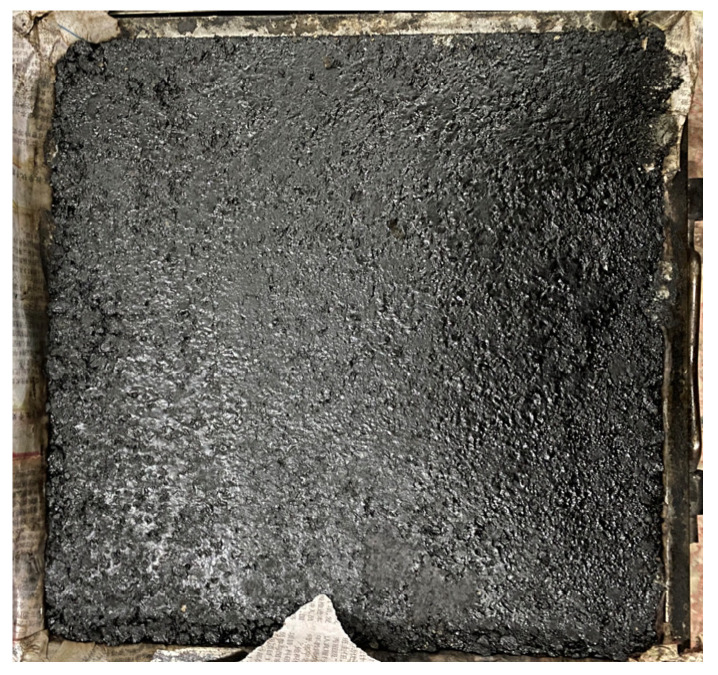
Specimen of the wheel tracking test.

The wheel tracking test is simple to operate and correlates with pavement rutting, which can truly reflect the mixture’s stress characteristics. Therefore, it is widely used to assess the asphalt mixture’s high-temperature stability. The wheel tracking test was used to analyze and research the asphalt mixture’s pavement performance by Shanghai Changji SYD-0719B automatic rutting tester in this research. The rut board’s size is 300 mm × 300 mm. The test temperature was 60 °C. The wheel pressure was 0.7 MPa. The test was conducted following the AASHTO T324-04 and T 0719-2011. The Figure 3 is the specimen of the wheel tracking test.

#### 2.3.5. Low-Temperature Stability

Asphalt mixture is a kind of temperature-sensitive material, and temperature changes will cause significant changes in the asphalt mixture’s mechanical properties. The low-temperature crack resistance means that the asphalt mixture has a particular strength and deformation performance at low temperature. In this study, a three-point loading method of laboratory trabecular specimens was used to research the asphalt mixture’s low-temperature stability performance by MTS-Landmark, Eden Prairie, MN, USA. The trabecular specimen’s size was 250 mm × 30 mm × 35 mm, the loading rate was 50 mm/min, and the experiment temperature was −10 °C.

#### 2.3.6. Water Stability

In this study, the asphalt mixture’s water stability was mainly evaluated by the freeze–thaw splitting test by MTS-Landmark and the Marshall test by Shanghai Changji SYD-0709A Marshall Stability Tester. According to the specifications, the Marshall specimens were formed. The Marshall test specimens were compacted 75 times on both sides and soaked for 48 h. The Marshall specimens of the freeze–thaw splitting test were compacted 50 times on both sides. The temperature of the unfreeze–thaw specimen was 25 °C. The loading rate was 50 mm/min. The test method after the freeze–thaw cycle adopted AASHTO T 283 and T 0729-2000.

## 3. Results and Discussion

### 3.1. Performances of the CMA

#### 3.1.1. Physical Properties

**Figure 4:**
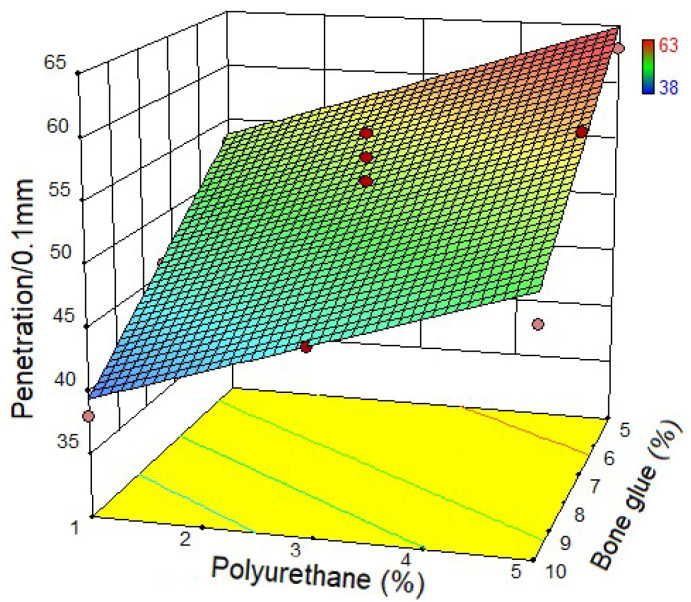
Penetration of the composite modified asphalt (CMA).

**Figure 5:**
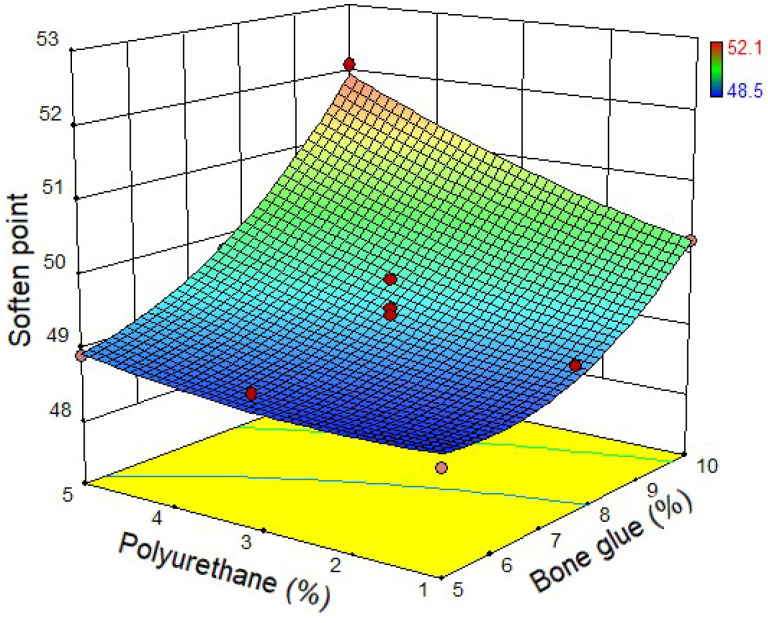
Soften point of CMA.

**Figure 6:**
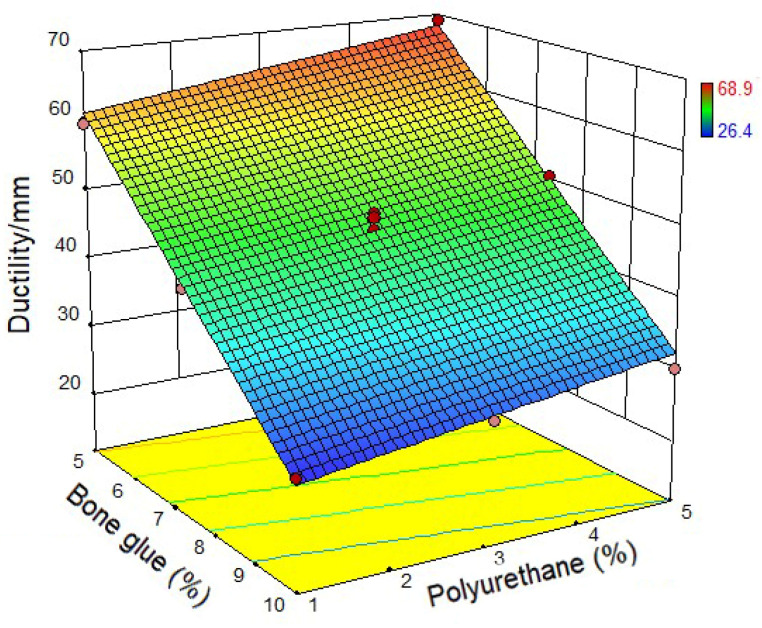
Ductility of composite modified asphalt.

Based on the test design, 13 groups of CMA were prepared. The physical properties test of CMA was conducted according to Section 2.3. The test results were shown in Figure 4, Figure 5 and Figure 6. Figure 4 shows the relationship between the penetration of CMA and bone glue and polyurethane content. It can be found that when the content of bone glue is the same, the penetration of CMA increases with the increase of polyurethane content. When the polyurethane content is the same, the penetration of CMA decreases with the increase of bone glue content. The results show that bone glue can harden the CMA and improve the high-temperature deformation resistance of asphalt. Figure 5 shows the relationship between the softening point of CMA and bone glue and polyurethane content. It can be seen that, when the content of bone glue is low, with the increase of polyurethane content, the softening point of CMA has no change. However, when the polyurethane content is fixed, with the increase of bone glue content, the softening point of CMA increases, which proves that the addition of bone glue significantly improves the high-temperature performance of CMA. Figure 6 shows the relationship between CMA’s ductility and the content of bone glue and polyurethane. The ductility at 5 °C was used to characterize the low-temperature deformation resistance of asphalt. It can be noticed that when the bone glue content is determined, the ductility of the CMA increases with the increase of polyurethane content. When the polyurethane content is the same, the ductility decreases with the increase of bone glue content. To sum up, polyurethane can enhance the low-temperature deformation resistance of asphalt.

#### 3.1.2. Creep Stiffness

In this study, the creep stiffness (*S*) and the change rate of asphalt stiffness under load (*m*) obtained from the BBR test were selected to assess the low-temperature performance of CMA. According to the American SHRP, the pavement is prone to crack under low-temperature conditions, manifested as the *S* of asphalt is larger, and the *m* value is smaller. The excellent low-temperature performance of asphalt materials is that the larger the *m* value, the greater the stress relaxation performance. The smaller the *S* value, the better the low-temperature flexibility.

**Figure 7:**
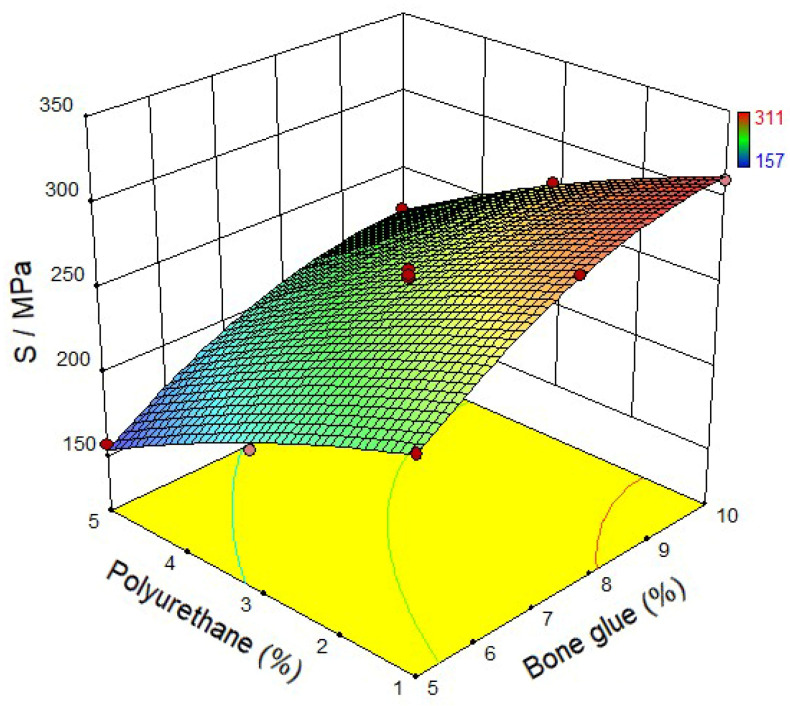
Stiffness modulus of CMA.

**Figure 8:**
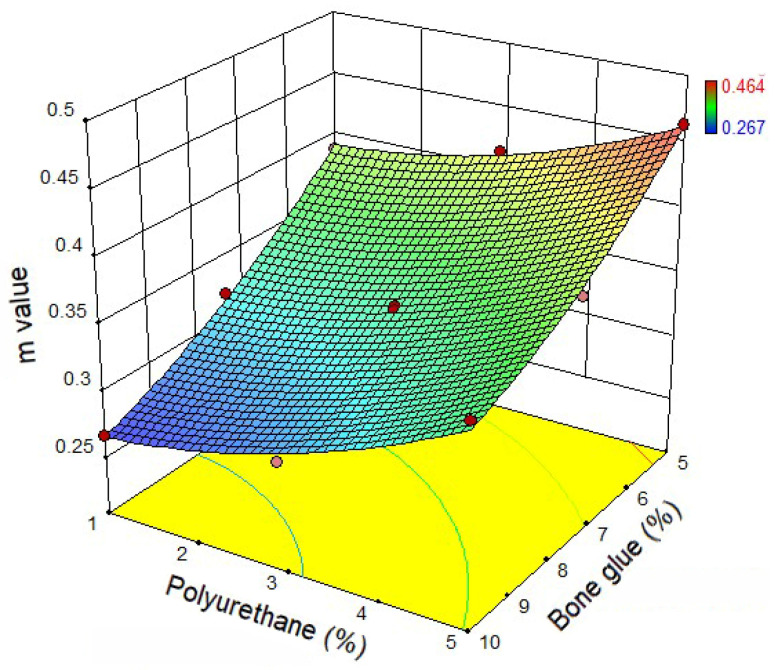
*m* value of CMA.

According to the BBR test results, it can be noticed from Figure 7 that when the polyurethane content is fixed, the *S* value gradually increases with the increase of bone glue content. The result represents that the content of bone glue in the CMA has an apparent influence on the low-temperature crack resistance of the composite system. From Figure 8, the *m* value gradually decreases with the increase of bone glue content at the same polyurethane content. Therefore, it can be inferred that the self-healing ability and low-temperature ductility of CMA after fatigue damage is reduced, mainly due to the gradual increase of bone glue content in the CMA. When the bone glue content is fixed, the *m* value of CMA with 5% polyurethane content is higher than that with 1% polyurethane content. The results show that polyurethane’s addition can improve the *m* value of the CMA and reflect that most of the internal stress generated in the CMA can be effectively absorbed and dispersed by polyurethane when an external force deforms it [21]. Then the low-temperature deformation resistance of the composite system is significantly enhanced.

#### 3.1.3. Non-Recoverable Creep Compliance

**Figure 9:**
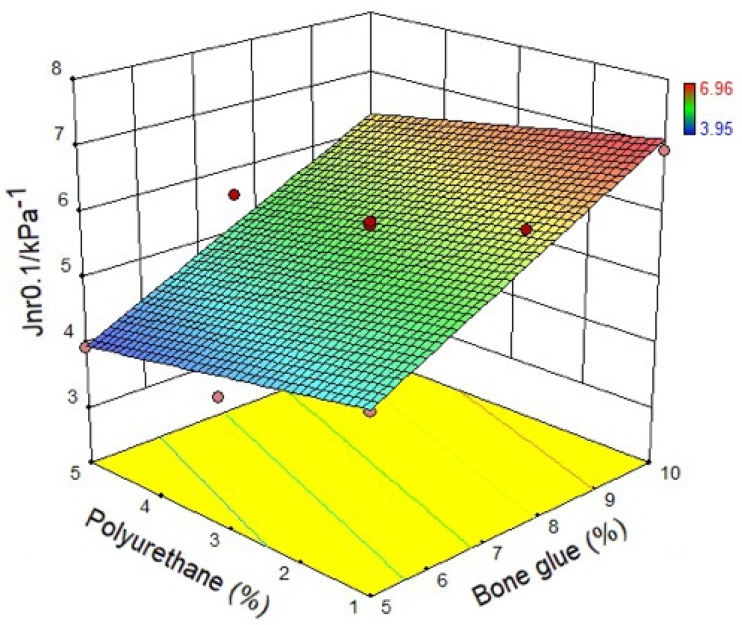
Non-recoverable creep compliance at 0.1 kPa.

**Figure 10:**
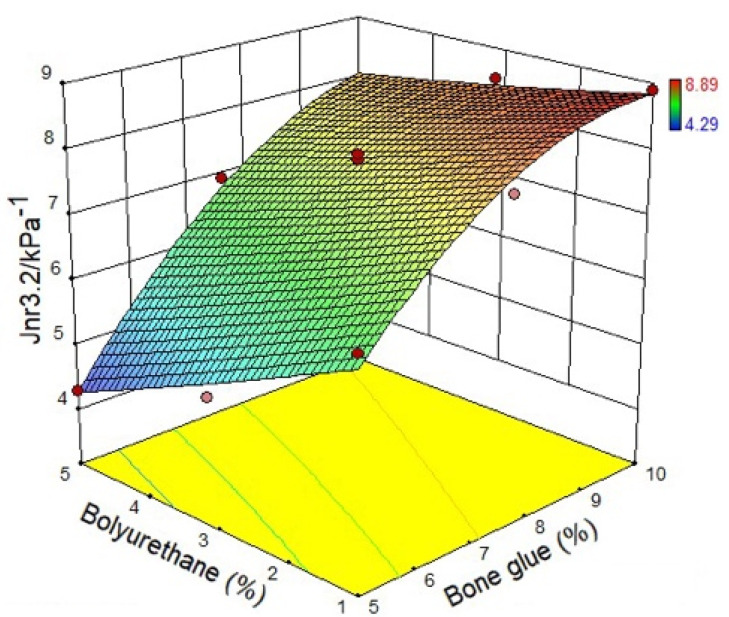
Non-recoverable creep compliance at 3.2 kPa.

In this study, the non-recoverable creep compliance corresponding to loading stress of 0.1 kPa and 3.2 kPa are recorded as *J_nr_*_0.1_ and *J_nr_*_3.2_, respectively. It can be noticed from Figure 9 and Figure 10 that the non-recoverable creep compliance decreases with the increasing of polyurethane content, which indicates that the high-temperature permanent deformation resistance of the CMA is strengthened by adding polyurethane. When the polyurethane content is the same, the non-recoverable creep compliance will increase correspondingly with bone glue content. This result expresses that polyurethane weakens the ability of the permanent deformation resistance of the CMA. In summary, it can be found that polyurethane can enhance the resistance to permanent deformation of asphalt.

### 3.2. Comprehensive Evaluation of Performance of CMA

In order to better determine the optimal content of bone glue and polyurethane, this study conducted a comprehensive evaluation based on each performance index. Therefore, based on the experimental results, the model was established for the physical performance and rheological properties of the CMA, which can better reflect the relationship between the CMA performance and the content of bone glue and polyurethane. Equations (1)–(7) are the penetration, softening point, ductility, stiffness modulus, creep rate, the non-recoverable creep compliance models under 0.1 kPa and 3.2 kPa of the CMA in sequence.

(1) Pen=52.15−7.17A+5.50B

(2) $$Sp=49.24+1.13A+0.5B+0.37AB+0.56A^{2}+0.16B^{2}$$

(3) Du=46.89−17.85A+3.47B

(4) $$S=255.69+36.17A-43.83B-0.25AB-16.91A^{2}-5.91B^{2}$$

(5) $$m=0.34+0.059A+0.039B+0.003AB+0.013A^{2}+0.015B^{2}$$

(6) $$J_{nr0.1}=5.56+1.18A-0.40B$$

(7) $$J_{nr3.2}=7.51+1.57A-0.71B+0.33AB-0.65A^{2}+0.037B^{2}$$

where *Sp* is the softening point (°C); *Pen* is the penetration at 25 °C (0.1 mm); *Du* is the ductility at 5 °C (mm); *S* is the stiffness modulus (MPa); *m* is the creep rate; *J_nr_*_0.1_ and *J_nr_*_3.2_ are the non-recoverable creep compliance under 0.1 kPa and 3.2 kPa, respectively; *A* is the content of bone glue, and *B* is the content of polyurethane. It can be noticed that the penetration, ductility, and non-recoverable creep compliance under 0.1 kpa are linear models, while other performance indicators are quadratic models. The results of each performance index are analyzed by variance, which is shown in Table 5.

From Table 5, the fitting correlation coefficient *R*^2^ is greater than 0.85. The content of bone glue and polyurethane dramatically influences the physical properties of the CMA. The results of multiple variances show that the established model has high accuracy. The models can effectively predict the performance of CMA according to independent variables. By using Design expert 10.0.4 to predict the performance of CMA based on the established model, the prediction results are shown in Table 6 and Table 7. It can be found that the maximum relative error between the test results and the model prediction results is 10.67%, which proves the high reliability of the model.

In summary, it can be concluded that when the content of bone glue is 6.848% and the content of polyurethane is 2.759%, the performance of CMA is the best based on the above model. At this level, the performance predicted results are as follows: penetration is 53.4 mm, softening point is 48.9 °C, ductility at 5 °C is 51.1 mm, stiffness modulus is 250.29 MPa, creep rate is 0.352, the non-recoverable creep compliance under 0.1 kPa and 3.2 kPa is 5.296 kPa^−1^, and 7.149 kPa^−1^, respectively. Based on the optimal content of bone glue and polyurethane determined by the model, the CMA was prepared with a shear time of 40 min, a shear rate of 3000 r/min, and a shear temperature of 145 °C. The performance test results of the CMA with the best content are shown in Table 8. As can be seen from Table 8, the predicted values of the model are accurate. The maximum relative error between the test value and the predicted value is 5.68%. In order to better verify the performance of CMA with the optimal content, the asphalt mixture’s pavement performance is further verified.

### 3.3. Pavement Performance of the CMA Mixture

#### 3.3.1. Determination of Optimum Asphalt Aggregate Radio

According to the target proportioning and bulk relative density, the bulk relative density of synthetic wool was obtained. The relationship between the bulk relative density of synthetic gross volume and the optimum asphalt aggregate radio of similar projects were analyzed. It was concluded that the optimum asphalt aggregate radio is 5.0%. Finally, the asphalt aggregate ratio of 4.0%, 4.5%, 5.0%, 5.5%, and 6.0% are selected to prepare the standard Marshall specimens. By testing the physical and mechanical indexes of the specimen, the Marshall test results are shown in Table 9.

According to the relationship between asphalt aggregate ratio in Table 1 and physical and mechanical indexes of Marshall specimen, the optimum asphalt aggregate ratio of bone glue/polyurethane CMA is 5.2%. By adopting the same method as the optimum asphalt aggregate ratio of CMA mixture, that of the neat asphalt mixture and SBS modified asphalt mixture can be determined as 4.7% and 5.0%, respectively. The neat asphalt is the Liaohe A-70. SBS modifier is a linear polymer 791H.

#### 3.3.2. High-Temperature Stability

In this research, a wheel tracking test was carried out according to relevant regulations, and dynamic stability is used to assess the asphalt mixture’s high-temperature performance. The wheel tracking test results are shown in Table 10.

It can be noticed that the dynamic stability of the CMA mixture is 85% of SBS modified asphalt mixture, but it is 2.4 times that of neat asphalt mixture. It concluded that the high-temperature stability of bone glue/polyurethane CMA mixture is higher than that of neat asphalt mixture. Although there are some gaps between CMA mixture and SBS modified asphalt mixture, these indicate that the CMA mixture still has certain advantages of high-temperature stability.

#### 3.3.3. Low-Temperature Stability

In this research, the mixture’s low-temperature performance was studied by the three-point loading method of the laboratory trabecular specimen. The test results are shown in Table 11.

It can be seen from Table 11 that the flexural tensile strength of bone glue/polyurethane CMA mixture is 1.46 times that of neat asphalt mixture, and 1.25 times that of SBS modified asphalt mixture. The test results show that the flexural tensile strength of CMA mixture is improved to some extent. The maximum bending tensile strain of the CMA mixture is 1.94 times that of the neat asphalt mixture, and 1.28 times that of SBS modified asphalt mixture. The test results show that the CMA has good flexibility. Therefore, the low-temperature performance of bone glue/polyurethane CMA mixture is better than that of neat asphalt mixture and SBS modified asphalt mixture.

#### 3.3.4. Water Stability

**Figure 11:**
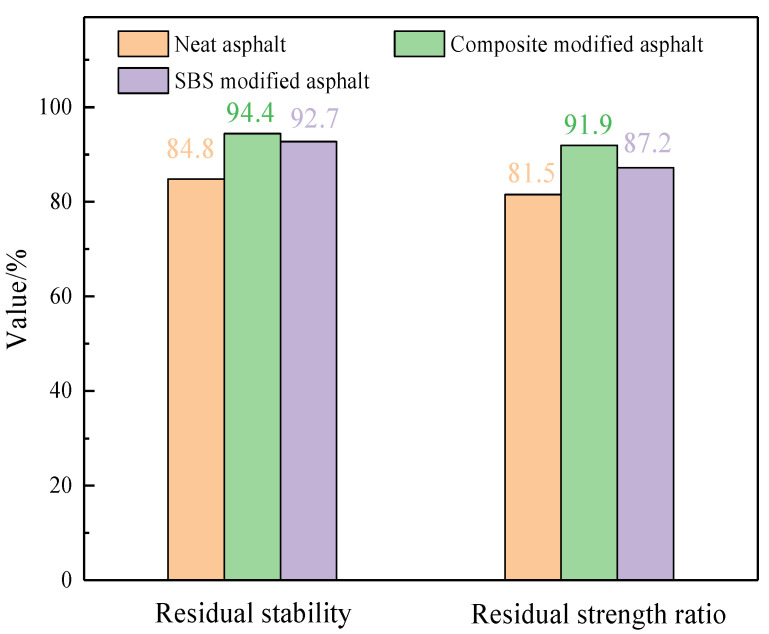
Test results of water stability.

The immersion Marshall test and freeze–thaw split test were carried out according to the test procedures. The test results are shown in Figure 11.

It can be seen from Figure 11 that the residual stability of bone glue/polyurethane CMA mixture is 1.11 times that of the neat asphalt, and 1.02 times that of SBS modified asphalt mixture. It indicates that the CMA mixture has good residual stability. Bone glue/polyurethane CMA mixture has better freeze–thaw performance than neat asphalt mixture and SBS modified asphalt mixture. Combined with the two indexes, bone glue/polyurethane CMA mixture has good water stability.

## 4. Conclusions

In this research, the response surface method was used to optimize the bone glue/polyurethane CMA performance. The CMA mixture’s pavement performance was investigated, and that of the neat asphalt and SBS modified asphalt mixture was compared. The main conclusions drawn are as follows:

(1) The penetration, softening point, ductility, multiple stress creep test, and bending beam rheometer test was conducted. The influence of the content of bone glue and polyurethane on the CMA was studied. It concluded that bone glue/polyurethane CMA could effectively improve the high-temperature and low-temperature performance of asphalt.
(2) The relationship between the performance indicators and modifiers’ content was revealed based on the test results. The fitting correlation coefficient of the model is greater than 0.85. The maximum relative error between the test results and the model prediction results is 10.67%. When the content of bone glue is 6.848%, and the polyurethane content is 2.759%, the performance of CMA is the best.
(3) The pavement performance of neat asphalt mixture, SBS modified asphalt mixture, and bone cement/polyurethane CMA mixture were compared and analyzed. It is found that the low-temperature crack resistance and water stability of the CMA mixture are far superior to the other two asphalt mixtures. The CMA mixture’s dynamic stability is 85% of the SBS modified asphalt mixture but is 2.4 times that of the base asphalt mixture, which shows that the CMA mixture has also improved high-temperature stability.
(4) In this paper, the properties of bone glue/polyurethane CMA and its mixture were studied. The optimal content of bone glue and polyurethane were determined. However, the modification mechanism of CMA and the durability of CMA mixture have not been studied yet. In order to better apply bone glue/polyurethane CMA in road engineering, the two aspects of research and the leaching tests will be carried out further.

## Figures and Tables

**Table 1:** Test results of neat asphalt.

Technical Index	Test Results	Technical Standard
Penetration at 25 °C/0.1 mm	67.8	60–80
Softening point (°C)	46.6	≥46
Ductility at 10 °C/cm	42	≥20
Ductility at 15 °C/cm	110	≥100
Density at 15 °C/g/cm^3^	1.029	-

**Table 2:** Test results of the polyurethane.

Technical Index	Test Results
Density (g/cm^3^)	1.11
Tear strength (N/mm^2^)	68
Hardness/Shore A	88
Tensile strength (N/mm^2^)	46

**Table 3:** Aggregate density test results.

Size (mm)	Apparent Relative Density (g/cm^3^)	Bulk Volume Relative Density (g/cm^3^)	Water Absorption (%)
16–13.2	2.562	2.581	1.24
13.2–9.5	2.654	2.572	1.53
9.5–4.75	2.648	2.585	1.35
4.75–2.36	2.640	-	-
2.36–1.18	2.635		
1.18–0.6	2.602		
0.6–0.3	2.588		
0.3–0.15	2.576		
0.15–0.075	2.609		

**Table 4:** Contents of bone glue and polyurethane.

Number	Bone Glue Content (%)	Polyurethane Content (%)
1	7.5	3
2	7.5	3
3	7.5	1
4	5	3
5	7.5	3
6	7.5	3
7	5	5
8	5	1
9	10	1
10	10	5
11	7.5	3
12	7.5	5
13	10	3

**Table 5:** Variance analysis results of the composite modified asphalt (CMA) performance.

Index	*Pen*	*Sp*	*Du*	*S*	*m*	*J_nr_*_0.1_	*J_nr_*_3.2_
Model	Linear	Quadratic	Linear	Quadratic	Quadratic	Linear	Quadratic
*R* ^2^	0.8595	0.9222	0.9870	0.9934	0.9929	0.8861	0.9610
Adjustment. *R*^2^	0.9314	0.8666	0.9844	0.9887	0.9878	0.8634	0.9331
Coefficient of Variation.%	5.42	0.74	3.45	1.81	1.62	6.22	4.67
Model F-value	30.59	16.59	378.49	210.12	194.81	38.92	34.47
Lack-of-Fit *p* value	0.8872	0.8056	0.5425	0.3468	0.0952	0.5852	0.7429
Model *p*-value	<0.0001	0.0009	<0.0001	<0.0001	<0.0001	<0.0001	<0.0001
Press	117.28	2.92	47.95	771.37	0.002	1.83	2.81
Standard Deviation	2.83	0.37	1.62	4.43	0.005	0.35	0.34

**Table 6:** Comparison of test results and model prediction results.

No.	*Pen*			*Sp*			*Du*			*S*		
	Test	Predicted	Relative Error (%)	Test	Predicted	Relative Error (%)	Test	Predicted	Relative Error (%)	Test	Predicted	Relative Error (%)
1	58	52.15	10.09	48.7	49.24	−1.11	46.9	46.89	0.02	258	255.69	0.90
2	49	52.15	−6.43	49.8	49.24	1.12	45.3	46.89	−3.51	261	255.69	2.03
3	46	46.65	−1.41	49.1	48.90	0.41	43.1	43.43	−0.77	295	293.61	0.47
4	58	59.32	−2.28	48.9	48.66	0.49	63.5	64.74	−1.95	198	202.61	−2.33
5	51	52.15	−2.25	48.9	49.24	−0.70	49.3	46.89	4.89	256	255.69	0.12
6	54	52.15	3.43	49.4	49.24	0.32	48.6	46.89	3.52	257	255.69	0.51
7	63	64.82	−2.89	48.9	48.94	−0.08	68.9	68.21	1.00	157	153.11	2.48
8	53	53.82	−1.55	48.5	48.69	−0.39	59.6	61.28	−2.82	241	240.28	0.30
9	38	39.49	−3.92	50.2	50.21	−0.02	26.4	25.58	3.11	311	313.11	−0.68
10	48	50.49	−5.19	52.1	51.96	0.27	30.2	32.51	−7.65	226	224.95	0.47
11	56	52.15	6.88	49.3	49.24	0.12	48.7	46.89	3.72	250	255.69	−2.28
12	59	57.65	2.29	49.8	49.90	−0.20	50.8	50.36	0.87	201	205.94	−2.46
13	45	44.99	0.02	50.8	50.93	−0.26	28.3	29.04	−2.61	276	274.94	0.38

**Table 7:** Comparison of test results and model prediction results.

No.	*m*			*J_nr_*_0.1_			*J_nr_*_3.2_		
	Test	Predicted	Relative Error (%)	Test	Predicted	Relative Error (%)	Test	Predicted	Relative Error (%)
1	0.342	0.34	0.47	5.02	5.56	−10.67	7.34	7.51	−2.27
2	0.335	0.34	−1.61	5.36	5.56	−3.65	7.96	7.51	5.70
3	0.321	0.32	1.28	6.48	5.95	8.12	8.02	8.26	−2.98
4	0.416	0.41	0.75	4.05	4.38	−8.08	5.07	5.28	−4.18
5	0.341	0.34	0.18	5.45	5.56	−1.93	7.02	7.51	−6.93
6	0.342	0.34	0.47	5.84	5.56	4.87	7.87	7.51	4.62
7	0.464	0.46	0.27	3.95	3.98	−0.73	4.29	4.28	0.31
8	0.389	0.39	−1.13	4.74	4.78	−0.75	6.56	6.36	3.03
9	0.267	0.27	0.10	6.96	7.13	−2.47	8.89	8.85	0.45
10	0.358	0.35	1.66	6.21	6.34	−2.02	7.93	8.07	−1.83
11	0.345	0.34	1.34	5.89	5.56	5.68	7.45	7.51	−0.76
12	0.387	0.39	−1.87	5.63	5.16	8.40	6.96	6.83	1.89
13	0.288	0.29	−2.16	6.64	6.73	−1.41	8.53	8.43	1.23

**Table 8:** Verification test results of CMA.

Index	*Pen*	*Sp*	*Du*	*S*	*m*	*J_nr_*_0.1_	*J_nr_*_3.2_
Unit	25 °C/0.1 mm	°C	5 °C/mm	MPa	-	kPa^−1^	kPa^−1^
Test results	54.2	49.1	52.3	236.84	0.358	5.186	7.039
Predicted results	53.4	48.9	51.1	250.29	0.352	5.296	7.149
Relative error (%)	1.48	0.41	2.29	−5.68	1.68	−2.12	−1.56

**Table 9:** Marshall test results of CMA mixture.

Asphalt Aggregate Radio (%)	4.0	4.5	5.0	5.5	6.0
Bulk density (g·cm^−3^)	2.418	2.440	2.472	2.488	2.475
Stability (kN)	17.31	18.42	18.94	18.60	18.16
Air void (%)	6.2	4.9	3.7	3	2.2
Flow value (mm)	2.01	2.75	3.10	3.52	4.21
Void ratio of mineral aggregate (%)	14.1	13.2	13.4	13.7	14.5
Saturation (%)	55.0	60.2	66.8	71.9	81.4

**Table 10:** Wheel tracking test results.

Type of Mixture	Rutting Depth at 60 min/mm	Dynamic Stability/Times·(mm^−1^)
Neat asphalt	4.308	1624
Bone glue/polyurethane CMA	3.041	3978
SBS modified asphalt	2.941	4630

**Table 11:** Low-temperature stability test results.

Type of Mixture	Maximum Load (N)	Maximum Bending Tensile Strain (×10^−3^)	Flexural Tensile Strength (MPa)	Mid Span Deflection at Failure (mm)	Bending Stiffness Modulus (MPa)
Neat asphalt	1002.48	2.15	7.52	0.4186	2489
Bone glue/polyurethane CMA	1391.69	4.18	10.98	0.8168	3648
SBS modified asphalt	1179.65	3.25	8.81	0.6248	2948

## Data Availability

The data is available on request from the corresponding author.
